# Accelerated subcutaneous nodulosis in patients with rheumatoid arthritis treated with tocilizumab: a case series

**DOI:** 10.1186/s13256-018-1687-y

**Published:** 2018-06-03

**Authors:** Rossella Talotta, Fabiola Atzeni, Alberto Batticciotto, Maria Chiara Ditto, Maria Chiara Gerardi, Piercarlo Sarzi-Puttini

**Affiliations:** 1 Department of Rheumatology, ASST Fatebenefratelli-Sacco, via GB Grassi n. 74, 20157 Milan, Italy; 2 Rheumatology Unit, University of Messina, via Consolare Valeria 1, 98100 Messina, Italy

**Keywords:** Rheumatic nodulosis, Tocilizumab, Rheumatoid arthritis

## Abstract

**Background:**

Tocilizumab is a monoclonal antibody directed against the interleukin-6 receptor, which is approved for the treatment of moderate-to-severe rheumatoid arthritis. Authors have found that it prevents lung and subcutaneous nodulosis in patients with rheumatoid arthritis but, to the best of our knowledge, there are no data concerning the acceleration of subcutaneous nodulosis during tocilizumab therapy.

**Case presentation:**

We report for the first time a small case series of five patients with rheumatoid arthritis: a 46-year-old white woman, a 70-year-old white woman, a 63-year-old white woman, a 69-year-old white man, and a 72-year-old white woman (mean age 64 ± 10.6 years); they experienced worsening subcutaneous nodulosis during treatment with intravenously administered tocilizumab.

Four of the five patients were positive for rheumatoid factor and five for anti-citrullinated peptide antibodies. All of the patients had previously been treated with various conventional and biological drugs; at the time of our observation, three were taking methotrexate, two hydroxychloroquine, and four were taking prednisone. Tocilizumab 8 mg/kg was administered intravenously every 4 weeks for a mean of 43.4 ± 32.4 months, and led to good disease control in three cases. All of the patients had a history of subcutaneous nodulosis, which considerably worsened during tocilizumab treatment, with the development of new nodules on their fingers, elbows, or in the inframammary fold, tending to ulcerate. The management of this medical event included discontinuation of methotrexate, the administration of steroids, the addition of hydroxychloroquine or colchicine, the use of antibiotics, and surgery. However, neither pharmacological nor surgical treatment was completely effective, as the nodules tended to recur and increased in number and size.

**Conclusions:**

To the best of our knowledge, this is the first report describing accelerated subcutaneous nodulosis in a small case series of patients with rheumatoid arthritis treated with tocilizumab.

## Background

Subcutaneous nodulosis describes an extra-articular manifestation occurring in patients affected by rheumatoid arthritis (RA). The nodules may develop in the subcutaneous layer of the hands and elbows, on Achilles tendons, and may also involve the lungs and vocal chords. Nodulosis may be accelerated by concomitant treatment with methotrexate (MTX) and, to some extent, azathioprine, leflunomide, and anti-tumor necrosis factor (TNF) agents, whereas the use of colchicine and hydroxychloroquine (HCQ) may improve the course of the disease.

The development of subcutaneous nodules in patients with RA has been explained on the basis of a rheumatic vasculitis, which is characterized by the precipitation of immune complexes in the small vessels and the subsequent activation of the complement cascade. Vessel inflammation is attributed to the infiltration of polymorphonuclear and mononuclear leukocytes, and the development of granulomas that are finally responsible for the formation of nodules.

Subtypes of RA characterized by high titers of rheumatoid factor (RF), other autoantibodies, or, more generically, a high degree of systemic inflammation are the most susceptible to extra-articular complications, including subcutaneous nodulosis. The use of biological drugs that interfere with the cytokines involved in systemic inflammation, such as interleukin (IL)-6, should be valuable in preventing the extra-articular manifestations of RA. Tocilizumab (TCZ) is a monoclonal antibody (moAb) directed against the IL-6 receptor that is approved for the treatment of moderate-to-severe RA, and some authors have found that it prevents lung and subcutaneous nodulosis in patients with RA [1, 2] but, to the best of our knowledge, there are no data concerning the acceleration of subcutaneous nodulosis during TCZ therapy.

## Case presentation

We describe a small case series of five white patients with RA (one man and four women; mean age 64 ± 10.6 years; mean disease duration 21.8 ± 10.9 years) who experienced significant worsening of subcutaneous nodulosis during treatment with intravenously administered TCZ. Patients were consecutively recruited from October 2016 to January 2017.

### Patient 1

The first case was a 46-year-old white woman who was nullipara, a non-smoker of tobacco, and unemployed. Since 1998 she had suffered from RF and anti-citrullinated peptide antibodies (ACPA)-positive and erosive RA associated with rheumatoid nodules; she had been treated with several conventional and biological drugs (MTX, sulfasalazine, leflunomide, HCQ, infliximab, etanercept, adalimumab, rituximab, certolizumab, abatacept) plus prednisone (at a stable dose of 7.5 mg/day throughout the years); all of the drugs were discontinued for inefficacy or adverse events. In June 2014 she started intravenously administered TCZ 8 mg/kg every 4 weeks plus MTX 10 mg/week (further increase in dose not tolerated) and HCQ 6 mg/kg a day, with minimal beneficial effects: C-reactive protein disease activity score on 28 joints (CRP-DAS28) was > 5.1 at the time of enrollment in the study. In 2006 (under etanercept therapy), anti-nuclear antibodies (ANA) turned positive to a 1.160 titer with a homogeneous pattern; anti-double stranded DNA (dsDNA) and anti-cardiolipin were always negative at follow-up. In 2010, anti-Ro SSA antibodies were detected despite no report of symptoms of an overlapping connective tissue disease. Rheumatoid nodules at the fingers of both her hands, which never showed a beneficial effect from all of the previous therapies, dramatically increased in size and number during TCZ treatment. Due to persistent high disease activity and the increase in the number and size of the rheumatoid nodules she was swapped to golimumab 50 mg every month in January 2017, without any further progression of subcutaneous nodulosis despite poor control of RA symptoms.

### Patient 2

The second case was a 70-year-old, white, menopausal, non-smoker of tobacco, retired woman. Since 1995 she had suffered from immunoglobulin M (IgM) RF-negative, ACPA-positive and erosive RA, and rheumatoid nodules. Since 1997 she received MTX 15 mg/week plus HCQ 400 mg/day, and in 2003, due to poor control of the disease, she underwent a treatment with intravenously administered infliximab 3 mg/kg every 8 weeks, which was discontinued in 2014 for a progressive lack of efficacy and the *de novo* detection of ANA and anti-dsDNA (appearing at the eighth infusion and undetectable at baseline). Subsequent biologic treatments with golimumab and abatacept showed no clinical effects; therefore, intravenously administered TCZ 8 mg/kg every 4 weeks combined with MTX 15 mg/week was started in June 2016. Since the start of TCZ, she experienced a progressive worsening of subcutaneous nodulosis in her hands, with nodules tending to cluster and ulcerate. Moreover, new ulcerating nodules appeared in her inframammary folds. An antibiotic treatment with amoxicillin/clavulanate acid 1000 mg/day for 6 consecutive days was prescribed in order to avoid infections. In December 2016 colchicine 1 mg every other day was added and MTX discontinued. However, subcutaneous nodulosis did not ameliorate, although no more ulcerations were reported.

At the time of enrollment (November 2016), RA disease activity was moderate (CRP-DAS28 4.79), and she was also taking prednisone 5 mg/day.

### Patient 3

The third case was a 63-year-old white woman who was menopausal, a tobacco smoker, and employed. She was RF and ACPA-positive, had rheumatoid nodules, and erosive RA was diagnosed in 1979 at another rheumatologic center. Since 2006 she attended our Department and started receiving orally administered MTX 7.5 mg/week and etanercept 50 mg/week administered by subcutaneous injection, both discontinued in 2009 for adverse events. Subsequently, she was treated with intravenously administered rituximab (discontinued for inefficacy), intravenously administered abatacept (discontinued for inefficacy), and, since April 2010, intravenously administered TCZ 8 mg/kg every 4 weeks in monotherapy (no compliance to conventional anti-rheumatic drugs), achieving and maintaining a good clinical response (CRP-DAS28 1.40 at the enrollment time). Subcutaneous nodules of her right elbow and fingers were pre-existent to the introduction of TCZ. However, 2 months later, she reported a worsening of subcutaneous nodulosis at the fingers of her left hand and at her right elbow, which underwent a central ulceration in February 2013. ANA and other auto-antibodies were negative at baseline and throughout the follow-up. A brief course of methylprednisolone 4 mg/day for 4 weeks and a preventive antibiotic therapy with amoxicillin/clavulanate acid 1000 mg/day for 6 days were prescribed, with some beneficial effects on ulcer healing.

### Patient 4

The fourth case was a 69-year-old white man who was retired and who smoked tobacco; he had suffered from RF and ACPA-positive, non-erosive RA since 2009. In 2009 he started a treatment with intravenously administered infliximab 3 mg/kg every 8 weeks plus orally administered MTX 7.5 mg/week and prednisone 2.5 mg/day with initial good disease control. In March 2010 his ANA titer was 1.160, with other autoantibody subsets negative. In March 2014 he was swapped to intravenously administered TCZ 8 mg/kg every 4 weeks due to progressive inefficacy and development of rheumatoid nodulosis. Concomitantly, a subcutaneous nodule of the first finger of his right hand was removed; the histologic diagnosis was compatible with a rheumatoid nodule. However, in April 2014, soon after the introduction of TCZ, he complained from the onset of a new subcutaneous ulcerated nodule at his left elbow. An antibiotic treatment with amoxicillin/clavulanate acid 1000 mg/day for 6 consecutive days was started. In July 2014, giving the benefit of TCZ in clinical disease activity and the risk of precipitating nodulosis, MTX was definitively discontinued. However, rheumatoid nodules at his fingers increased in number and, in November 2016, HCQ 200 mg/day was added. At the enrollment time (November 2016), RA disease activity was in remission (CRP-DAS28 2.1). No development of new autoantibody positivity or change in ANA titration was recorded.

### Patient 5

The last case was a 72-year-old white woman who was menopausal, a non-smoker of tobacco, and a housewife. In 1990 she was diagnosed as having erosive RF and ACPA-positive RA and rheumatoid nodules. Since then she received MTX 7.5 mg/week (further increase in dose not tolerated) plus prednisone 5 mg/day, with few improvements in symptoms, and subsequently several biologic drugs (infliximab, etanercept, rituximab, and adalimumab), which were all discontinued for adverse events or inefficacy. During follow-up, her ANA titers fluctuated from negative to positive values and vice versa (maximum titer recorded 1.160), whereas anti-dsDNA and other autoantibodies were persistently negative. In June 2010 she was considered a candidate for intravenously administered TCZ 8 mg/kg every 4 weeks, which resulted in good control of RA (CRP-DAS28 1.74 at the enrollment time, in November 2016), despite a mild leukopenia (absolute neutrophil count > 1000/mm^3^). From 2014 she also received HCQ 400 mg/day which was permanently discontinued in 2016 due to visual disturbances. She was affected by subcutaneous nodules at her elbows and fingers prior to the start of TCZ; she experienced a progressive increase in the size and number of the nodules during the treatment, and these manifestations partially worsened following the discontinuation of HCQ (not assumed at the enrollment time).

Table 1 shows the patients’ demographic characteristics.

**Table 1:** Demographic characteristics of the five patients with rheumatoid arthritis

Patients	*n* = 5
Gender (F/M), *n*	4/1
Age (years), mean ± SD	64.0 ± 10.6
Disease duration (years), mean ± SD	21.8 ± 10.9
Patients RF-positive, *n* (%)	4 (80%)
Patients ACPA-positive, *n* (%)	5 (100%)
Patients ANA-positive, *n* (%)	4 (80%)
Patients anti-dsDNA-positive, *n* (%)	1 (20%)
TCZ treatment duration (months), mean ± SD	43.4 ± 32.4
Patients treated with prednisone (2.5–7.5 mg/day), *n* (%)	4 (80%)
Patients treated with methotrexate (7.5–15 mg/week), *n* (%)	3 (60%)
Patients treated with hydroxychloroquine (200–400 mg/day), *n* (%)	2 (40%)

Demographic characteristics of the five patients included in the study. Four of the patients were positive for rheumatoid factor and five for anti-citrullinated peptide antibodies; four patients had anti-nuclear antibodies and one had anti-double-stranded DNA antibodies. All of the patients had previously been treated with various conventional and biological drugs; at the time of observation, three were taking methotrexate at a mean dose ± SD of 11.6 ± 3.8 mg/week with folic acid supplementation; two were taking hydroxychloroquine at a dose of 300 or 400 mg/day; and four were taking prednisone 2.5–7.5 mg/day. The patients had also been treated with intravenously administered TCZ 8 mg/kg every 4 weeks for a mean ± SD of 43.4 ± 32.4 months. *ACPA* anti-citrullinated peptide antibodies, *ANA* anti-nuclear antibodies, *dsDNA* anti-double stranded DNA, *F* females, *M* males, *RF* rheumatoid factor, *SD* standard deviation, *TCZ* tocilizumab

## Discussion

We report five cases of accelerated subcutaneous nodulosis under intravenously administered TCZ 8 mg/kg every 4 weeks for a mean 43.4 ± 32.4 months of treatment.

**Fig. 1:**
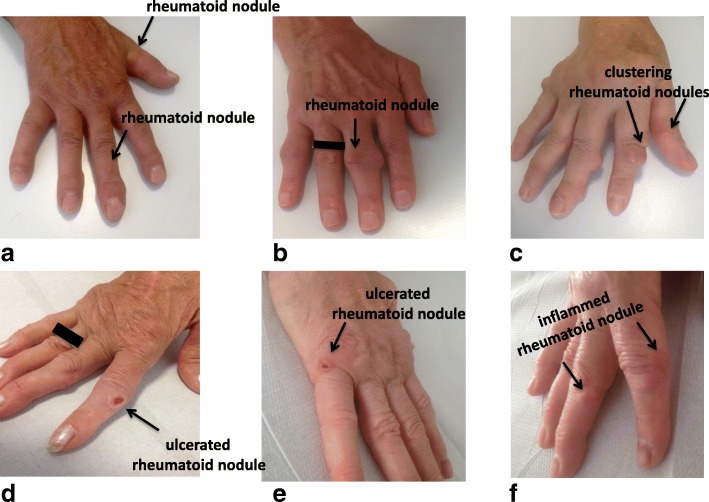
Subcutaneous nodules on the dorsal surface of the hands of five patients with rheumatoid arthritis treated with tocilizumab. The nodules tended to cluster and ulcerate (**c**–**e**), and were surrounded by an erythematous halo (**f**) that was associated with local pain and disability

All the patients had a history of subcutaneous nodulosis, which considerably worsened during treatment with TCZ. They developed new subcutaneous nodules mainly on the fingers (Fig.  1a-f), but also on the elbow or in the inframammary fold. The nodules had a tendency to ulcerate and released a serous but not purulent fluid, and perilesional inflammation and local pain greatly affected their quality of life.

As there are still no standardized guidelines, this medical event was managed in various ways, including the tapering or discontinuation of MTX (two cases), the administration of steroids (one case), the addition of HCQ or colchicine (one case), the use of antibiotics to prevent infective complications (three cases), and surgery with a histological examination that revealed a granulomatous pattern compatible with rheumatoid nodules (one case). However, neither pharmacological nor surgical treatment was completely effective as the nodules tended to recur and increased in number and size. In one case, TCZ was permanently discontinued because of poor clinical efficacy; the patient was switched to an anti-TNF agent (golimumab) and is still being followed up.

Subcutaneous nodulosis is the most frequently encountered extra-articular manifestation of RA, and occurs in up to 35% of patients [3]. The nodules typically develop on the dorsal surface of the fingers, the elbows or Achilles tendons, although they may also involve the lungs or vocal chords, and are often associated with high RF or ACPA titers, and a more aggressive and erosive course of RA. A genetic substrate, cigarette smoking, and male gender have been controversially associated with the risk of developing nodulosis [4]. The nodules are usually characterized histologically by a granulomatous process, with a central area of fibrinoid necrosis and a peripheral layer of epithelioid macrophages, lymphocytes, and histiocytes. There is some evidence that the nodules may arise as a T helper (Th)1-mediated response, and highly express cytokines such TNF-α, IL-10, IL-15, IL-18, and IL-12 [5, 6] but, interestingly, IL-17 does not seem to be involved in their pathogenesis. Granulomatous responses are related to the activation of Th1 lymphocytes and macrophages favoured by the local production of cytokines such as IL-2 and TNF-α, whereas IL-17 seems to be mainly involved in lymph node organization and in the formation of germinal centers [7]; IL-17 is therefore associated with Th2 and B lymphocyte activation and antibody production that depend on a distinct immune pathway. By preventing the maturation of Th-17 lymphocytes and the subsequent release of IL-17, TCZ may shift the immunological balance toward a more pronounced Th1 response that indirectly promotes rheumatic nodulosis, although currently available data are contrasting [8–10].

The use of MTX (and sometimes azathioprine, leflunomide, and anti-TNF agents) may accelerate the growth of pre-existing nodules [11–14]. Drug-induced subcutaneous nodules have the same clinical and histological characteristics as RA-related nodules, but the mechanism by means of which the drugs favor subcutaneous nodulosis while controlling articular symptoms is still unclear. Genetic susceptibility has been described in patients carrying the polymorphic variants of methionine synthase reductase (MTR) or haplotype HLA-DRB1*0401 [15, 16], and some authors have demonstrated that treatment with MTX is associated with the reactivation of Epstein–Barr virus, which may be responsible for the development of lymphoproliferative disorders or subcutaneous nodules [17]. The concomitant use of colchicine, sulfasalazine, or HCQ seems to improve the course of the complication, and is therefore recommended in cases in which the discontinuation of MTX is contraindicated [11]. Anti-TNF-α biologic therapy (for example, infliximab and etanercept) is commonly used in the management of RA, especially in patients who are MTX-resistant. There have been reports of accelerated subcutaneous and lung nodulosis in patients with RA, developing early after starting infliximab or etanercept, perhaps as a result of new autoimmune phenomena triggered by the neutralization of TNF-α [12, 18].

To the best of our knowledge, there are no data concerning accelerated subcutaneous nodulosis during treatment with TCZ. Some reports have even highlighted the usefulness of TCZ in treating lung nodulosis induced by previous treatment with anti-TNF agents [1], and there is one case report indicating its beneficial effect on olecranon subcutaneous nodules in a patient with RA [2]. Given its biological properties, TCZ reduces the burden of systemic inflammation and prevents extra-articular manifestations, endothelial dysfunction, and vasculitis, but there are some published reports describing the occurrence of dermatological complications such as leukocytoclastic vasculitis and toxidermia [19, 20].

We have observed a visible worsening of subcutaneous nodulosis in 8.3% of the 60 patients receiving TCZ treatment at our center, which may be related to a change in the immune response toward more pronounced Th1 activation. In addition, in our cohort of patients treated with TCZ, there have also been three cases (5%) with subcutaneous nodulosis which, however, remained stable during the treatment. Further studies are required to clarify the mechanisms of pathogenesis underlying this association, and the real incidence of this little known event.

## Conclusions

In conclusion, we report for the first time five cases of accelerating subcutaneous nodulosis in patients with RA intravenously treated with TCZ. All five patients suffering from pre-existing rheumatic nodulosis experienced its severe progression with local inflammation and ulceration. Our data suggest that a clearer understanding of RA may allow the identification of subtypes not previously appreciated, finally leading to personalized therapies. However, given the apparently protective effect of TCZ on the extra-articular manifestations of RA, the association between its use and the development/worsening of subcutaneous nodulosis deserves further investigations.
